# Dr. A. Palmer Dudley

**Published:** 1905-08

**Authors:** 


### OBITUARY.

Dr. A. Palmer Dudley, of New York, one of the distinguished gynecologists of the country, died at Liverpool, England, July 15, 1905, of pulmonary tuberculosis, aged 52 years. Dr. Dudley attended the meeting of the American Gynecological Society at Niagara Falls, in May last, looking the picture of health. He was on his way to Saint Petersburg to attend the International Congress of Gynecology and Obstetrics as a delegate from the former society.

